# Neonatal Hyperglycemia Inhibits Angiogenesis and Induces Inflammation and Neuronal Degeneration in the Retina

**DOI:** 10.1371/journal.pone.0079545

**Published:** 2013-11-21

**Authors:** Elsa Kermorvant-Duchemin, Alexis Christophe Pinel, Sophie Lavalette, Delphine Lenne, William Raoul, Bertrand Calippe, Francine Behar-Cohen, José-Alain Sahel, Xavier Guillonneau, Florian Sennlaub

**Affiliations:** 1 INSERM UMRS872, Centre de Recherche des Cordeliers, Paris, France; 2 Paris Descartes University, Paris, France; 3 AP-HP, Necker-Enfants malades Hospital, Paris, France; 4 INSERM, U 968, Paris, F-75012, France; 5 CNRS, UMR_7210. Paris, F-75012, France; 6 Institut de la Vision, UPMC Univ Paris 06, UMR_S 968, Paris, France; 7 AP-HP, Hôtel Dieu, Service d'Ophtalmologie, Centre de Recherche ophtalmologique, Paris, France; 8 Centre Hospitalier National d'Ophtalmologie des Quinze-Vingts, INSERM-DHOS CIC 503, Paris, France; 9 Fondation Ophtalmologique Adolphe de Rothschild, Paris, France; University of Florida, United States of America

## Abstract

Recent evidence suggests that transient hyperglycemia in extremely low birth weight infants is strongly associated with the occurrence of retinopathy of prematurity (ROP). We propose a new model of Neonatal Hyperglycemia-induced Retinopathy (NHIR) that mimics many aspects of retinopathy of prematurity. Hyperglycemia was induced in newborn rat pups by injection of streptozocine (STZ) at post natal day one (P1). At various time points, animals were assessed for vascular abnormalities, neuronal cell death and accumulation and activation of microglial cells. We here report that streptozotocin induced a rapid and sustained increase of glycemia from P2/3 to P6 without affecting rat pups gain weight or necessitating insulin treatment. Retinal vascular area was significantly reduced in P6 hyperglycemic animals compared to control animals. Hyperglycemia was associated with (i) CCL2 chemokine induction at P6, (ii) a significant recruitment of inflammatory macrophages and an increase in total number of Iba+ macrophages/microglia cells in the inner nuclear layer (INL), and (iii) excessive apoptosis in the INL. NHIR thereby reproduces several aspects of ischemic retinopathies, including ROP and diabetic retinopathies, and might be a useful model to decipher hyperglycemia-induced cellular and molecular mechanisms in the small rodent.

## Background

Retinopathy of prematurity (ROP), is one of the major causes of visual impairment in children. It is characterized by an initial retardation of the progression of vascular growth toward the retinal periphery which predisposes to abnormal compensatory neovascularisation [1]. Low birth weight, low gestational age and supplemental oxygen therapy are the major risk factors for the development of ROP. In the very low birth weight infant, hyperglycemia occurs frequently, especially in the first weeks after birth [2], [3], [4], [5]. Elevated blood glucose concentration has been recognized as an additional and independent risk factor for ROP irrespective of the threshold used to define hyperglycemia (between 144 and 180 mg/dl) [6], [7], [8], [9], [10], [11]. Each 10 mg/dL increase of mean serum glucose has been shown to increase the risk of ROP 2.7 fold [9] and each additional day of hyperglycemia increase the risk to develop the disease by 7% [11].

In diabetic adults, adverse effects of poor glycemic control and hyperglycemic state on retinal vasculature and function are well-known [12]. The features and mechanisms of diabetic retinopathy (DR) have been extensively studied in spontaneously diabetic and streptozotocin-induced retinopathy in adult rodents. The effects of hyperglycemia on the developing retina remain unknown.

The objectives of this work were to characterize the effect of a moderate, clinically relevant, hyperglycemic phase on vascular development. Our results show that neonatal hyperglycemia very reproducibly induces severe inflammation, inhibition of physiological angiogenesis and neuronal degeneration in a very short period of time. Neonatal hyperglycemia retinopathy (NHIR) might be useful to help decipher hyperglycemia-induced cellular and molecular mechanisms in the retina that lead to diseases such as ROP and DR.

## Methods

### Ethics

All experimental protocols and procedures were approved by the local animal care ethics committee “Comité d'éthique en expérimentation animale Charles Darwin” (N° p3/2008/54) and met the INSERM guidelines.

### Animals

One day-old Lewis rat pups were used for the experiments. Pregnant animals were purchased from Janvier - Le Genest- St-Isle, France. Animals were housed at local animal facilities under 12/12 hours light/dark cycles and fed ad libitum. The number of pups was culled to 8–10 per litter at P1 by random selection.

### Model of Neonatal Hyperglycemia

The model of neonatal hyperglycemia was adapted from previously published studies [13], [14], [15] using streptozotocin (STZ) (Sigma-Aldrich Chemical Co, Saint-Quentin Fallavier, France). For each experiment, aliquots of STZ were pre-weighed, wrapped in aluminium foil and stored in −20°C. STZ was dissolved in the citrate buffer (20 mmol/l) immediately before the injection to a final concentration of 10 mg/ml. 50 mg/kg (5 µl/g), was administered via intraperitoneal injections in the lower left quadrant of the abdomen, using a 29G needle (total volume injected = 25 to 30 µl depending on individual animal's weight). Control (CTL) animals received an equal volume of citrate buffer. The pups were returned to their dams after the injection. Before, and daily after STZ injection, animals were weighted and tail blood was collected for glucose determination using a standard patient glucometer (Accu-Chek® Performa, Roche Diagnostics, Meylan, France). Insulin blood levels were determined by ELISA using Mercodia Rat Insulin Assay (Mercodia SAS, Paris, France) according to manufacturer recommendations. Some rat pups were injected intravitreally with 1 µl of 1 mg/ml STZ at P3 to assess its toxicity on the developping retina.

### Immunohistochemistry

Pups were sacrificed serially by decapitation at P3, P4, P5, P6 and P7 (2 to 6 days after the STZ injection). Some rat pups were also assessed at P21, when the maturation of the retina is complete and the hyperglycemia has resolved. After sacrifice, eyes were removed and retinas were dissected and fixed in 4% paraformaldehyde for 20 minutes at room temperature prior to flatmounting or OCT (Tissue Tek®) embedding. Intraretinal vascularization and microglial cell density were assessed on whole retinal flatmounts. Retinal capillaries were labeled according to previously described standard immunohistochemical procedures [16], using goat polyclonal anti-collagen IV antibody (1/400, AbD Serotec, Cergy Pontoise, France) and Isolectin B4-FITC (Ib4) (Sigma Aldrich Chemical Co, Saint-Quentin en Yvelines, France). Pericytes were stained using NG2 antibody (1/200, Millipore, Saint-Quentin Fallavier, France) and microglia was labeled using rabbit polyclonal anti-Iba1 (1/400, Wako Pure Chemical Industries, Neuss, Germany). The corresponding Alexa–conjugated secondary antibodies (Invitrogen, Cergy Pontoise, France) were used to reveal the primary antibodies.

OCT-embedded retinas were cryosectioned and sections through the optic nerve were used for immunolabeling. Frozen sections were stained according to previously described standard procedures. In addition to the antibodies used on retinal flatmounts, the following primary antibodies were used to study retinal cells type on cryosections: anti-Ap2α antibody (3B5, for amacrine and horizontal cells) was purchased to Developmental Studies Hybridoma Bank (DHSB, Iowa city, Iowa); mouse anti-glutamine synthetase antibody (MAB302, for Muller cells) was obtained from Millipore (Saint-Quentin Fallavier, France), and rabbit anti-protein kinase Cα antibody (PKCα) (Sc-208, for bipolar cells) from Santa Cruz (Heidelberg, Germany). Anti-calretinin antibody (AB5320, Millipore, Saint-Quentin en Yvelines, France) was used to label horizontal cells, and anti-Rho4D2 (kind gift of Dr D. Hicks) to label rods. GFAP antibody (Sigma-Aldrich, Saint Quentin Fallavier, France) was used to label astrocytes. The corresponding Alexa–conjugated secondary antibodies (Invitrogen, Cergy Pontoise, France) were used to reveal the primary antibodies. The FITC conjugated-peanut agglutinin (PNA) was used to label cone photoreceptor (Sigma Aldrich Chemical Co, Saint-Quentin en Yvelines, France). Sections were counterstained with DAPI.

### Assessment of retinal vascularization and quantification of microglial cell density

Sections and flatmount images were captured with a DM5500 microscope (Leica, Nanterre, France) equipped with an ORCA ER Hamamatsu camera and analyzed by MetaMorph software (Molecular Devices, Saint-Gregoire, France). For each retina, tube length and branching of capillaries, and vascular density were measured on a minimum of 6 representative images (1 to 2 mm^2^) using the “angiogenesis tube formation” MetaMorph add-in (Molecular Devices, Saint-Gregoire, France). Parameters were set to select vessels between 3 and 40 µm. Vascularized areas were measured and expressed as percent of total retinal area [16]. Iba1-positive cell body size, cell perimeter (length around the periphery of each cell), process length (average distance from the cell body to its detected extremes) were measured on flatmounts using MetaMorph software (Molecular Devices, Saint-Gregoire, France). Cell roundness was calculated as described by Kozlowski et al [17].

### Histology/assessment of neurons layers

Pups exposed to hyperglycemia and their controls were sacrificed at P14 (when neuronal development and differentiation are at their end in rodent species), and retinas were immediately dissected and fixed in 0.5% glutaraldehyde, 4% paraformaldehyde PBS for 2 hours, dehydrated, and mounted in HistoResin. Sagittal, oriented sections (5 µm), crossing the inferior pole, optic nerve, and superior pole were cut (ultramicrotome Reichert Ultracut E, Leica, Nanterre, France), and stained with toluidin blue. Sections were photographed with a light microscope (Leica, Nanterre, France) and numbers of rows of the outer nuclear layer as well as number of the nuclei of the inner nuclear and ganglion cell layers were quantified at different distances from the optic nerve to the periphery.

### Reverse Transcription and Real-Time Polymerase Chain Reaction

Pups exposed to hyperglycemia and their normoglycemic controls were sacrificed at P6. Retinas were dissected and total RNA was extracted using the Nucleospin RNAII Kit (Macherey-Nagel, Hoerdt, France). Retrotranscription was performed using superscriptII (Invitrogen, Cergy-Pointoise, France). Real-time PCR was performed using 7300 Real-Time PCR System (Applied Biosystems, Cergy-Pointoise, France) in a 20 µl final volume with Power SYBR Green PCR Master Mix (Applied Biosystems, Cergy-Pointoise, France) and 0.25 µM primers. All samples were run in triplicate. Primers used for real-time PCR analysis are available upon request.

### Statistical analysis

All data are reported as mean +/− SEM unless otherwise indicated. Data were analyzed by unpaired t-test or one- and two-way ANOVA with Bonferroni post-tests, according to the number of groups compared (GraphPad Software Inc., San Diego, USA). *p*<0.05 was considered as statistically significant.

## Results

### STZ leads to transient hyperglycemia without growth retardation in newborn rats

**Figure 1:**
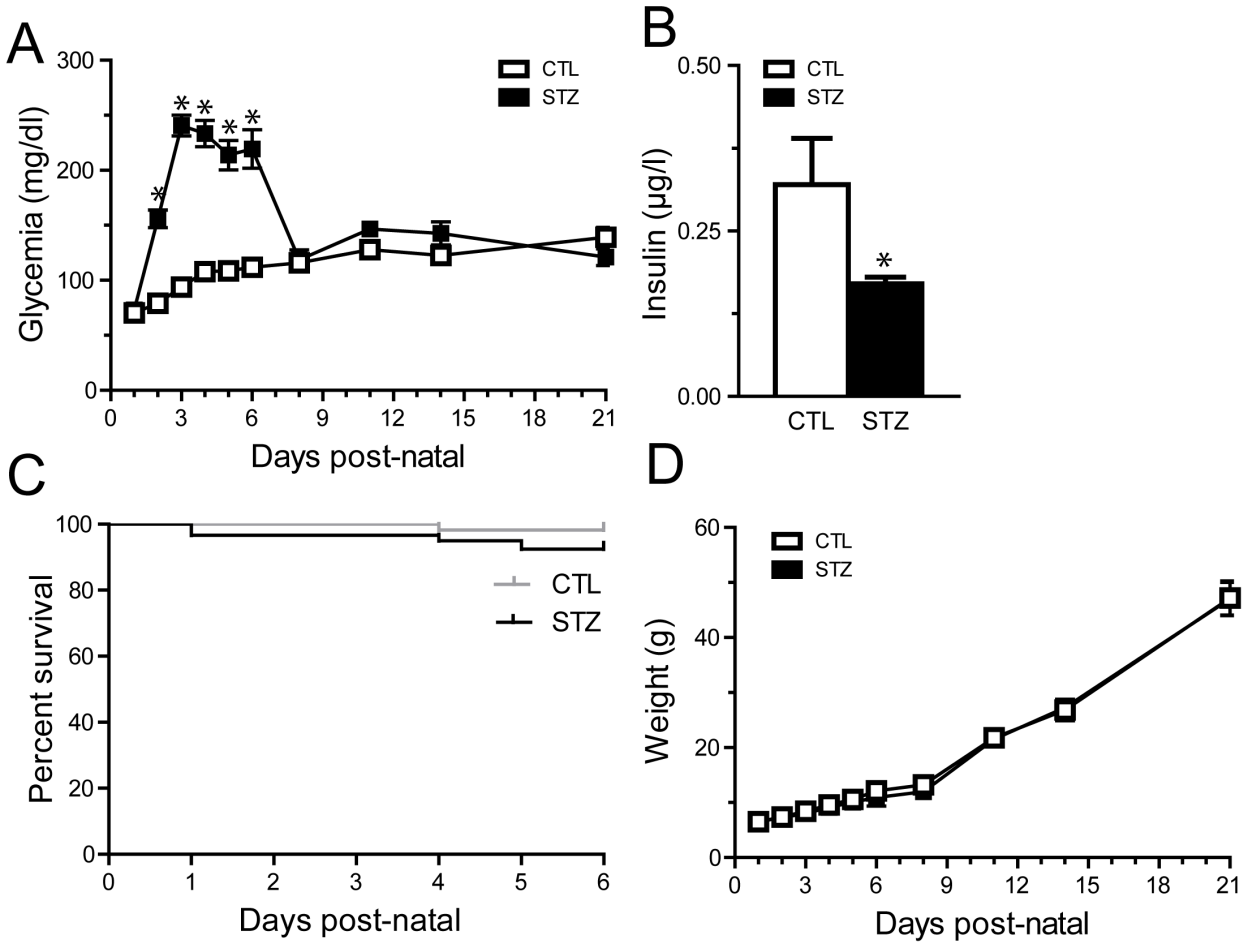
STZ leads to transient hyperglycemia without growth retardation in neonates. P1 rat pups were injected with low doses of streptozotocin in citrate buffer (STZ) or with the control vehicle (citrate buffer, CTL). **A**. Measurements of glycemia from P1 to P21 in both groups. The STZ group displayed a moderate increase in glycemia from P3 to P6, averaging 214 to 241 mg/dl (11.9–13.4 mmol/l). **B**. Insulin concentration in serum at P6 in control (white) and STZ treated (black) animals. The STZ group demonstrated a decreased level of insulin. **C**. Survival curve in STZ- and CTL-groups. Mortality was similar in both groups. **D**. Weight from P1 to P21 in STZ- and CTL-groups. Weight gain was not affected in STZ-injected animals when compared to control animals. Data in A and D were analyzed by a two-way ANOVA followed by a Bonferroni post test. Data in B were analyzed by an unpaired t-test. Data in C were analyzed by a Log-rank test. * P<0.05.

Hyperglycemia was induced by low STZ dose intraperitoneal injection at ages during which both the neural retina and the retinal vasculature are immature [18], [19]. Pilot studies determined 50 mg/kg as the lowest dose of STZ that induced sustained elevated glycemia without inducing life-threatening hyperglycemia. All animals were injected at postnatal day 1 (P1) with STZ (50 mg/kg) in citrate buffer (STZ group) or with the control vehicle (citrate buffer, CTL group). Administration of STZ resulted in a moderate increase of blood glucose concentration reaching 155 mg/dl (8.6 mmol/l) within 24 h following injection. Glycemia was significantly increased from P3 to P6, averaging 214 to 241 mg/dl (11.9–13.4 mmol/l) (Fig. 1A). At P6, values in STZ-injected pups ranged from 104 to 496 mg/dl with a mean value of 219.4+/−17.59 SEM (n = 51). A few animals experienced glycemia above 400 mg/dl between P3 and P6 (n = 3). The values in STZ-injected pups were not significantly different from controls beyond P7 (average CTL, 121.9 mg/dl, n = 14; average STZ, 136.8 mg/dl, n = 14, two-way Anova followed by Bonferroni post-test) (Fig. 1A). To monitor the efficiency of STZ-induced pancreatic Langerhans cell death we determined insulin blood levels in control and STZ animals. At P6, after 3 consecutive days of hyperglycemia, STZ injection resulted in a significant decrease in circulating insulin in STZ animals when compared to control group (0.32+/−0.07; n = 10 vs 0.17+/−0.01; n = 15, unpaired t-test, p = 0.02) (Fig. 1B). As decreased levels of insulin may affect survival of newborn animals we determined animal survival. Survival was minimally impacted by STZ and hyperglycemia and no significant difference was observed in death rate between the control and the STZ group following injection (Fig. 1C). Weight gain was not affected in hyperglycemic animals when compared to normoglycemic control animals (Fig. 1D).

**Figure 2:**
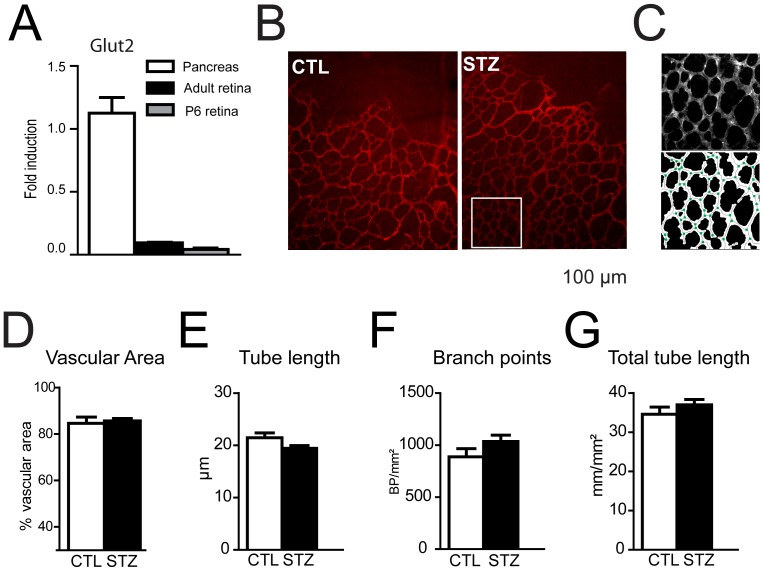
Intravitreal injection of STZ does not modify retinal vasculature. **A**. Glucose transporter GLUT-2 expression (RT-PCR) in newborn and adult retinas, as compared to adult pancreas. GLUT-2 expression was similar in P6 and adult retina but 22.6 fold lower than in adult pancreas. **B–F**. P1 rat pups were injected intravitreously with streptozotocin (STZ) or with the citrate buffer vehicle (CTL). **B**. Retinal flatmounts of P6 CTL or STZ animals were stained using anti-collagen IV antibody. **C** Representative automated analysis of vascular density using Metamorph software. Branch points are represented by green points and tubes by solid gray lines (lower panel). **D–G**. Vascular area, mean tube length, branch point density and total tube length density were determined at P6 in hyperglycemic (black bars) or control (white bars) animals. Values in histograms D–G are mean ± SEM of retinas from 8 animals per group from at least 2 different experiments. Data were analyzed by unpaired t-tests. No significant differences were found in these parameters.

STZ toxicity is dependent on glucose transporter GLUT2, through which it enters the cell. GLUT2 is strongly expressed on Langerhans cells [20]. It is not expressed on adult retinal neurons [21] and STZ has no direct toxicity on the mature retina [22], [23], [24]. To evaluate direct STZ susceptibility of the developing retina we assayed GLUT2 expression by qPCR during retinal development and compared it to adult pancreas and retina. P6 and adult rat retina GLUT2 expression was similar and 22.6 fold lower than in adult rat pancreas (Fig. 2A). We next assayed toxicity of STZ to retinal vasculature by injecting STZ directly into the vitreous (IVT) of newborn rats at P1. IVT injection neither affected weight gain nor glycemia (data not shown). Retinal vasculature was stained at P6 using an anti-collagen IV antibody in CTL and STZ animals (Fig. 2B). A software analysis tool was used (Fig. 2C) to determine, the vascularized retinal area (Fig. 2D), the mean tube length of the vessels (Fig. 2E), the number of branch point per mm^2^ (Fig. 2F) and the total tube length per mm^2^ (Fig. 2G). These parameters were not affected by STZ IVT injection, demonstrating an absence of vasculature toxicity of STZ in the newborn retina (p>0.05, unpaired t-test).

### Retinal angiogenesis is inhibited in hyperglycemic animals

**Figure 3:**
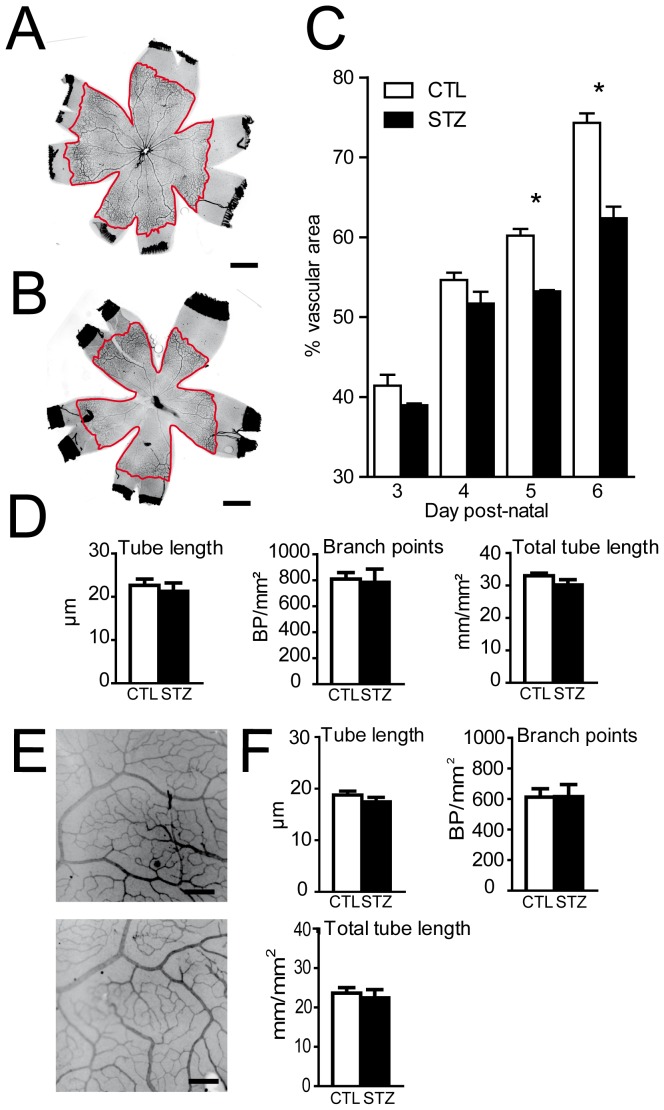
Retinal angiogenesis is inhibited in hyperglycemic animals. P1 rat pups were injected with low doses of streptozotocin (STZ) or with the citrate buffer vehicle (CTL). **A–B**. Retinal flatmounts of P6 CTL (A) or STZ (B) animals were stained using anti-collagen IV antibody. Vascularized area circumferences are highlighted in red. **C**. Vascular area measurement in hyperglycemic (black bars) or control (white bars) animals. Values in histograms are mean ± SEM of vessel area of retinas from 4–8 animals per group from 3 different experiments. * *P*<0.05 compared to CTL, two-way ANOVA, Bonferroni post-test. **D**. Mean tube length, branch point density and total tube length density were determined at P6 in hyperglycemic (black bars) or control (white bars) animals. Values in histograms are mean ± SEM of retinas from 4–8 animals per group from 3 different experiments. **E**. Higher magnification of P21 retinal flatmounts of CTL and STZ animals stained with collagen IV antibody. **F**. Mean tube length, branch point density and total tube length density were determined at P21 in hyperglycemic (black bars) or control (white bars) animals. Values in histograms are mean ± SEM of retinas from 4 animals per group from 2 different experiments. Scale bar 1 mm in A–B; 200 µm in E–F. No statistical differences in vessel parameters were found in D and F between STZ and control groups using unpaired t-tests.

Prolonged periods of hyperglycemia induce pericyte and endothelial cell death resulting in microvascular degeneration in the adult [25]. To evaluate the effect of moderate hyperglycemia on retinal vascular development we visualized the basal membrane of the retinal vasculature using an anti-collagen IV antibody at different time points of the hyperglycemic phase (Fig. 3A and B illustrate P6 vasculature in control and hyperglycemic animals respectively) and quantified retinal vascularization (Fig. 3C). While the vascularized retinal area was similar in control and STZ-treated animals at P3 and P4, a significant decrease in vascular area was found at P5, after 2 days of hyperglycemia (9.2%+/−1.8 (n = 6)) (Fig. 3C). At P6, inhibition of vascularization reached 15.6%+/−1.8 (n = 16) (Fig. 3A–C). Tube length, branch point density and total tube length per mm^2^ was determined at P6 in control and STZ groups to evaluate vessel density. No significant differences were found between groups (Fig. 3D). Similar results were obtained with the Ib4 lectin that stains viable endothelial cells (data not shown). The total retinal size in control and STZ injected animals at P6 was not significantly different (average CTL 30.0 mm^2^, average STZ 28.4 mm^2^, n = 30, unpaired t-test, p = 0.53). At P21, vessel density of the superficial plexus was evaluated. No differences were observed between control and STZ retinas (Fig. 3E–F).

**Figure 4:**
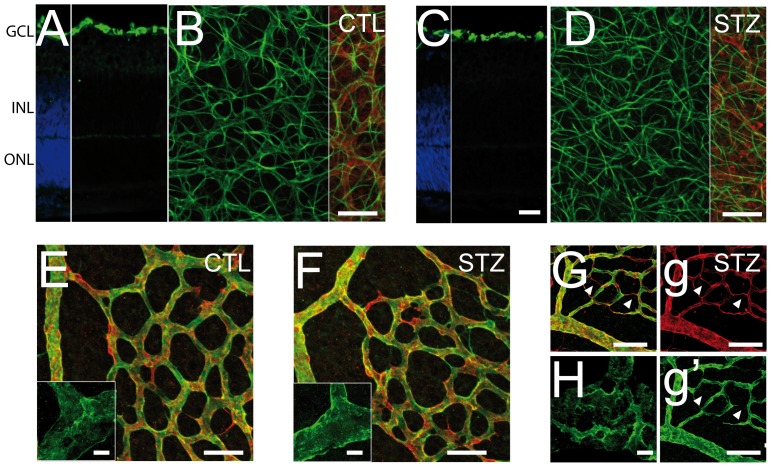
Astrocytes and pericytes phenotype in NHIR. P1 rat pups were injected with low doses of streptozotocin (STZ) or with the citrate buffer vehicle (CTL). **A–D**. Retinal flatmounts and sections of P6 CTL (A–B) and STZ (C–D) were co-stained using anti-collagen IV antibody and anti-GFAP antibody. **E–F** Retinal flatmounts of P6 CTL (E) and STZ (F) were co-stained using anti-collagen IV antibody and or anti-Ng2 antibody. **G–H**. STZ animals with hyperglycemia >400 mg/DL were co-stained using anti-collagen IV antibody and anti-Ng2 antibody. Nuclei were counterstained with DAPI in A and C. Scale bar 50 µm in A–G, g and g′; 5 µm in E and F insets and H.

Astrocytes enter the developing retina from the brain along the developing optic nerve during the first 10 postnatal days of rat retinal development [26]. GFAP positive astrocytes were correctly located in the GCL and no sign of reactive gliosis was detected in Müller cells in the hyperglycemic animals (Fig. 4A, C). When observed on retinal flatmount at P6, astrocyte processes bundle in contact with the vessels in the vascularized area giving rise to a more vascular pattern (Fig. 4B). In the hyperglycemic animals, astrocyte cell processes in the vascularized area stayed evenly distributed at the surface of the retina regardless of the vasculature pattern resembling their appearance in the non-vascularized retina at P6 (Fig. 4D). Vessel coverage by pericytes was evaluated using pericyte marker NG2 (Fig. 4E–H). Similar to control animals, NG2 staining in STZ-injected animals was found throughout the retinal vasculature up to the vascular front at P6 (data not shown). Higher magnification analysis of the vasculature did not reveal any signs of pericyte coverage loss (Fig. 4E–F) nor pericyte retractions (Fig. 4E–F insets) in animals with hyperglycemia around 214 to 241 mg/dl. Interestingly signs of pericyte coverage loss, capillary loss (Fig. 4G, g, g′, arrows), and pericyte retraction (Fig. 4H) were found in 3 animals that developed glycemia above 400 mg/dl.

### Neuronal and Muller cell genesis in hyperglycemic animals

**Figure 5:**
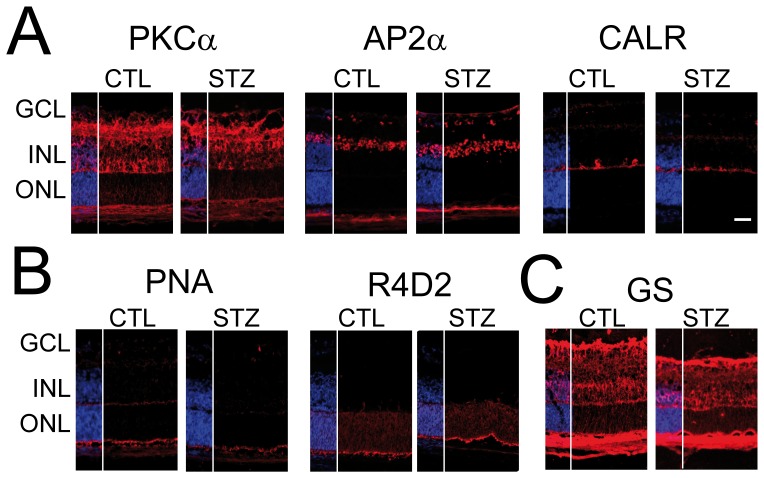
Neuronal and Muller cell genesis in hyperglycemic animals. **A–C**. Retinal sections of CTL and STZ-treated P6 rat pups were stained with various antibodies specific to neurons of the INL (A), photoreceptors cells (B) and glial cells (C) of the retina. **A**: INL neuron generation was not affected by hyperglycemia and similar patterns of staining (red) were observed in CTL and STZ animals for PKCα (bipolar cells), Ap2α (amacrine cells) and calretinin (CALR, horizontal cells). **B**. Photoreceptor generation was not affected by hyperglycemia and similar patterns of staining (red) were observed for peanut agglutinin (PNA, cones), Rho4D2 (R4D2, rods) in STZ P6 animals compared to CTL. **C**. Muller cells cell genesis was not affected by hyperglycemia when compared to CTL. Nuclei were counterstained with DAPI. Scale bars 50 µm. GCL = ganglion cell layer; INL = inner nuclear layer; ONL = outer nuclear layer.

Retinal neurons and Müller glial cells develop from a common pool of progenitors from E14 to P10 [19]. To evaluate if hyperglycemia blocks the generation of a particular retinal neuron or macroglia, we analyzed the expression of retinal specific markers of the inner nuclear layer (INL) and the outer nuclear layer (ONL) by immunohistochemistry in the mid peripheral retina at P6. Positive cells for PKCa (on bipolar cells), Ap2a (amacrine cells) and calretinin (horizontal cells) (Fig. 5A) were generated and correctly located in the INL of both group. PNA positive (cones) and Rho4D2 (rods) (Fig. 5B) photoreceptors were present in CTL and STZ P6 animals. Müller cells are the last cell type to be differentiated from retinal progenitors. There was no obvious difference in glutamine synthetase staining (Müller cells) of both the STZ and control, suggesting that Müller cells are generated in both groups similarly (Fig. 5C). Hyperglycemia did not block the generation of a specific cell type in particular.

### Hyperglycemia induces apoptosis and retinal degeneration

**Figure 6:**
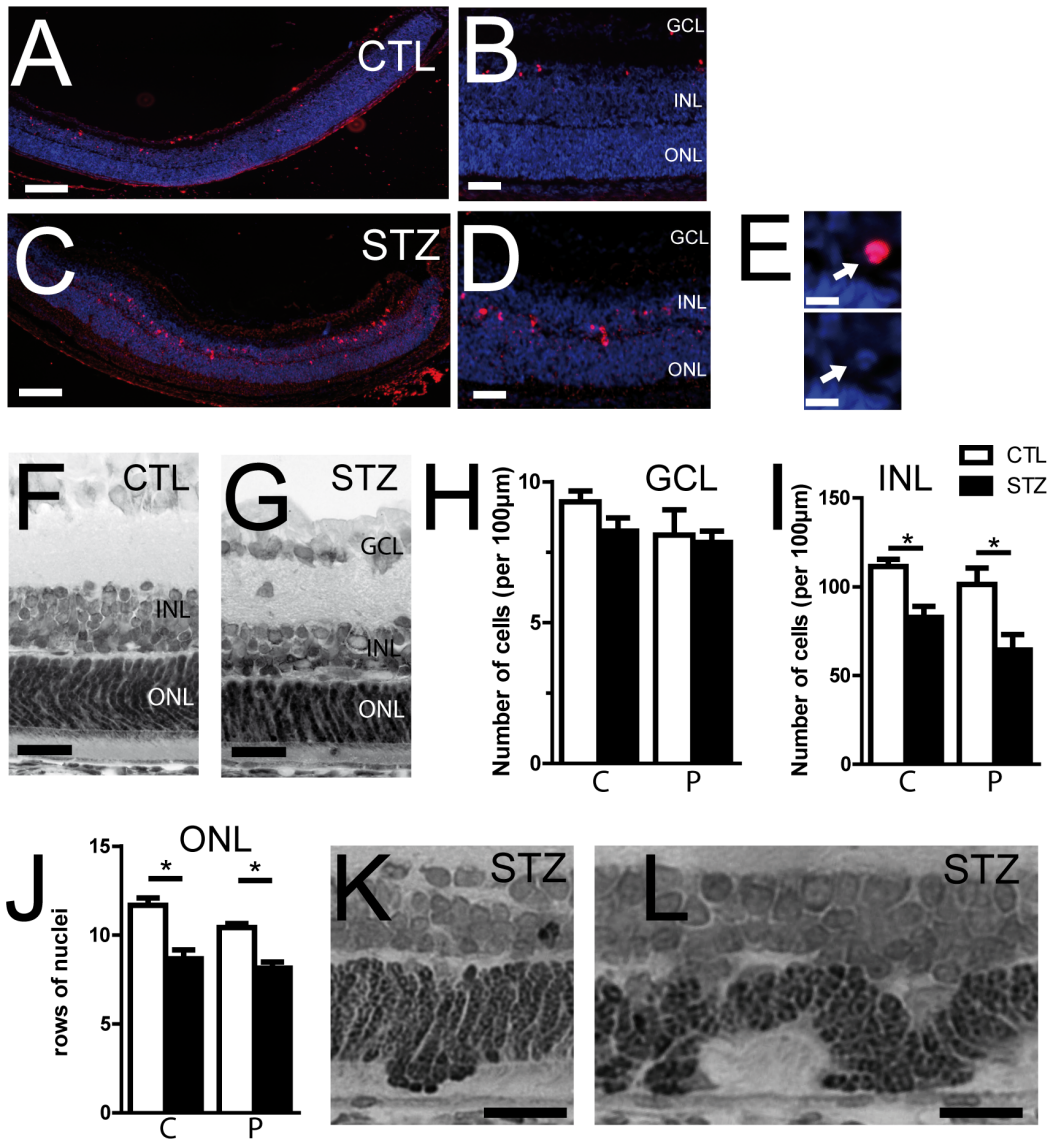
Hyperglycemia induces apoptosis and retinal degeneration. **A–D**. Retinal sections of STZ (C, D) and CTL (A, B) P6 animals were stained with the TUNEL labeling kit (red) and counterstained with DAPI. TUNEL labeling revealed the presence of apoptotic and fragmented nuclei in the INL of STZ retina (E). **F–G**. Representative histological toluidin-blue stained retinal sections in CTL (F) and STZ (**G**) P14 rat pups. **H–I**. Quantification of the number of nuclei per 100 µm in the central part (C) and the periphery (P) of the ganglion cell layer (H) or the INL (I), in hyperglycemic (black bar) and normoglycemic P14 animals (white bar). **J**. Quantification of the number of row of photoreceptor nuclei in the central part (C) and the periphery (P) of the retina, in hyperglycemic (black bar) and normoglycemic P14 animals (white bars). Values are mean +/− SEM of 9–10 retinas from 2 different experiments (H; I; J). * p<0.05; one-way ANOVA followed by Bonferroni post-tests. The number of nuclei in the INL and ONL was significantly reduced in STZ animals compared to CTL; this effect was predominant at the periphery of the retina. **K–L**. Representative histological toluidin-blue stained retinal sections of P14 STZ rat pups showing advanced structural disorganization with rosettes (K) and folds in the outer nuclear layers (L). Scale bars: 100 µm in A and C; 50 µm in B and D; 5 µm in E; 20 µm in F, G, K and L. GCL = ganglion cell layer; INL = inner nuclear layer; ONL = outer nuclear layer.

ROP leads to long-term impairment of retinal function [27] and diabetes induces neuronal apoptosis and degeneration in patients and in adult diabetic animal models [28], [29], [30]. To examine apoptosis in hyperglycemia induced retinopathy we performed TUNEL staining (red staining) on P6-old control (Fig. 6A–B) and hyperglycemic animals (Fig. 6C–D). In normoglycemic animals some TUNEL positive nuclei can be observed in the periphery of the GCL, while no or little apoptosis is observed in the INL and ONL. In contrast, hyperglycemic rat pups presented numerous TUNEL positive nuclei in the INL and ONL (fig. 6C–D). Higher magnification of TUNEL positive nuclei showed additional morphological signs of apoptosis such as fragmentation (Fig. 6E). Co-immunostaining with cell-specific markers did not allow the identification of a particularly affected cell type in the INL (data not shown). To evaluate if the observed increased apoptosis leads to permanent alteration of the retinal morphology, we compared toluidin blue-stained retinal sections of control (Fig. 6F) and hyperglycemic animals (Fig. 6G) at P14, when retinal stratification is physiologically mature. Nuclei of the GCL (Fig. 6H), the INL (Fig. 6I) and ONL (Fig. 6J) were quantified in hyperglycemic (STZ) and normoglycemic animals (CTL). While we did not observe differences in the GCL (measured as the number of cells per 100 µm), the numbers of nuclei of the INL (measured as the number of cells per 100 µm) and ONL (quantified as the number of rows) were significantly diminished in the STZ group. Nuclear layers in the central retina were less affected. In some hyperglycemic animals, more severe alterations of the outer nuclear layer were also seen, such as aberrant cells protruding into the subretinal space (Fig. 6K) and large folds of the ONL (Fig. 6L).

### Hyperglycemia leads to retinal inflammation

**Figure 7:**
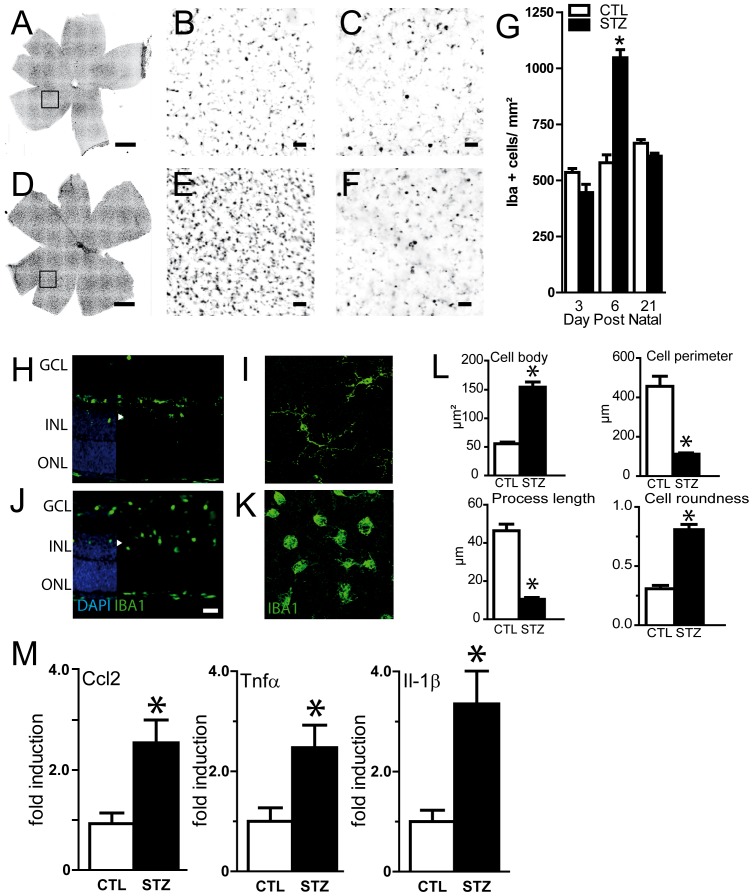
Effect of neonatal hyperglycemia on macrophage/microglial cells (MP/MC) recruitment. **A–F**. Retinal flatmounts and sections were stained with Iba-1 antibody. Representative flatmounts and sections of control (A, B) and STZ (D, E) P6 animals or P21 animals (C, F). **G**. Quantification of Iba-1 positive cells at different time points in CTL (white bars) and STZ (black bars) animals. MC recruitment peaked at 5–6 days postnatal. Values are mean +/− SEM. *p<0.05 Two-way ANOVA, Bonferroni post-test. **H–K**. Retinal sections and flatmounts of CTL (H–I) and STZ animals (J–K) were stained with Iba-1 antibody. MC/MP were located deeper in the retina of in hyperglycemic animals, reaching the inner nuclear layer (J) and displayed a change in their morphology, with round, bloated bodies and short ramifications indicative of an activated state (K). **L**. Cell body size, number of processes, length of the processes and perimeter length of Iba1 positive cells were determined for control (white bars) and hyperglycemic (black bars) animal at P6. Values in histograms are mean ± SEM of at least 50 cells selected in 3 different experiments. *p<0.05, Unpaired t-tests. **M**. Real-time PCR of *Ccl2*, *Tnfα* and *Il-1β* in P6 rat pups retinas exposed to hyperglycemia (STZ, black bars; n = 5) compared to controls (CTL, white bars, n = 5). *p<0.05, Unpaired t-tests compared to controls. Nuclei were counterstained with DAPI in H and J. Scale bars: 1 mm in A and D; 50 µm in B–C, E–F and H–K. GCL = ganglion cell layer; INL = inner nuclear layer; ONL = outer nuclear layer.

Microglial cells (MC)/macrophages (MP) have been shown to be activated in experimental and human diabetic retinopathy [31], [32], [33], [34], [35]. To evaluate MC/MP activation in neonatal hyperglycemia we labeled retinal flatmounts of control (Fig. 7A–C) and hyperglycemic pups (Fig. 7D–F) with the specific microglial cell/macrophage marker Iba1. Quantification of Iba1-positive MC/MP density at the beginning (P3), during (P6) and after hyperglycemia (P21) revealed a significant increase of retinal Iba1-positive cells by 80% in STZ injected pups at P6 (Fig. 7G and compare Fig. 7E and 7B) that renormalized at P21 after 14 days of renormalized glycemia (Fig. 7G and compare Fig. 7F and 7C). Iba1-stained retinal sections and flatmounts at P6 of control (Fig. 7H and I) and hyperglycemic pups (Fig. 7J and K) showed that retinal MC/MPs were physiologically mainly located in the GCL and IPL at P6 (Fig. 7H). Hyperglycemic animals additionally displayed many enlarged and rounded MC/MPs in the INL (Fig. 7J and K). To quantify the morphological signs of activation of the Iba1-positive cells, we analyzed the Iba1^+^ cells in the inner retina of control (Fig. 7I) and hyperglycemic rat pups (Fig. 7K). Iba1^+^ cells in hyperglycemic animals show an increase in the cell body size and cell roundness (Fig. 7L), a shortening of the cell ramification, and a decrease of their cell perimeter length (Fig. 7L). None of these parameters were affected by direct injection of STZ in the vitreous at P1 demonstrating an absence of direct role of STZ on Iba1^+^ cells activation (data not shown). Activated MC/MPs express inflammatory cytokines such as CCL2, TNFα, and IL-1β in experimental diabetes [32], [36], [37]. Real time RT-PCR analysis of mRNA extracts of P6 retina revealed a significant increase of *Ccl2* (2.54+/−0.45 vs 0.92+/−0.21), *Tnfα* (2.47+/−0.47 vs 1.00+/−0.27), and *Il-1β* (3.35+/−0.68 vs 1.00+/−0.60) in the STZ-injected hyperglycemic group compared to controls (n = 5, p<0.05, unpaired t-tests) (Fig. 7M). *iNos* was undetectable in both groups (data not shown).

## Discussion

Here we show that clinically relevant hyperglycemia during retinal development significantly inhibits retinal angiogenesis, induces neuronal cell death and leads to MC/MP infiltration and inflammatory cytokine production. Neonatal Hyperglycemia-induced retinopathy is an inexpensive, reproducible and quantifiable model that mimics retinopathy of prematurity.

Our goal was to develop a model of clinically relevant neonatal pathological hyperglycemia, with sustained levels of hyperglycemia at the time when angiogenesis and neuronal development take place in the retina [18], [19]. In a study by Hays et al., 42% of extremely low birth weight infants (birth weight <1000 g) had blood glucose concentrations consistently above 150 mg/dl (8.25 mmol/l) in the first week of life; for 6% of them, blood glucose concentrations were consistently above 250 mg/dl [4]. In the NIRTURE study, 33% of the very low birth weight infants (birth weight <1500 g) enrolled in the control group had glucose levels above >180 mg/dl (10 mmol/l) more than 10% of the time [2]. Our model reproduced hyperglycemia within ranges of those experienced by sick premature infants [2], [3], [4], [5] (Fig. 1).

We induced a moderate diabetic state with low mortality and little impact on body weight gain (Fig. 1), which is important since poor weight gain has been shown to be a risk factor for ROP in humans and in animal models [38]. Premature babies and type-1 diabetic patients are characterized by low insulin levels relative to their hyperglycemic state [39]. We here show that STZ injection in the neonate rat recapitulates hyperglycemia with low, but not null, levels of insulin. Insulin has neuroprotective effects [40] and the lack of insulin, as a neuro-trophic factor, encourages the loss of retinal neurons observed in diabetes [41] and possibly in hyperglycemic premature babies with ROP. We dosed the STZ so that our animals do not require insulin injections for survival, to avoid the potential neuro-trophic effects that would interfere with the model.

STZ is a widely accepted tool for examining the mechanisms of diabetic retinal injury and potential therapeutic interventions as shown by its extensive use in adult animals [42]. We here show that injection of STZ in the vitreous of newborn rats did not affect angiogenesis nor trigger inflammation in the retina (Fig. 2 and data not shown). Extrapancreatic effects of high doses of STZ have been shown in the liver and the kidney [22], [43]. It is possible that similarly STZ injection affects neonate organs. The unique 50 mg/kg injection used in our protocol compared to multiple 50–150 mg/kg injections in adult models possibly mitigated potential non-specific effects of STZ in pups. No edema or growth retardation was observed in STZ-treated pups, indicating that gross organ function was preserved and only the nenonates with glycemia >400 mg/l and above, (n = 3) were moderately icteric.

Elevated blood glucose concentration in the neonatal period has recently been recognized as an independent risk factor for development of ROP in very low birth weight infants [6], [7], [8], [9], [10], [11]. Garg et al. showed that for each 10 mg/dl increase of mean serum glucose, there was a 2.7-fold increase in the risk of developing ROP [9]. Blanco et al. also found that hyperglycemia >150 mg/dl was associated with a 4.6-fold increase in the incidence of ROP [6]. In a population of extremely low birth weight infants who did not receive insulin therapy, Mohamed et al. showed that infants with ROP experienced a greater mean number of days with hyperglycemia defined as whole blood glucose >150 mg/dl (7 days vs. 2 days, p = <0.0001); each day of hyperglycemia increased the risk of ROP by 7% [11]. We here show that 4 days of moderate hyperglycemia suffice to significantly inhibit the progression of the vascular ridge, reflected by a reduced vascularized retinal surface (Fig. 3) similar to ROP.

ROP leads to long-term impairment of retinal function [27] and diabetes induces neuronal apoptosis and degeneration in patients and in adult diabetic animal models [28], [29], [30]. Similarly, NHIR resulted in a decreased thickness of the peripheral INL and ONL at P14 when the retinal layering is mature (Fig. 6). We have observed an un-physiological apoptosis in the peripheral INL and in the ONL (Fig. 6). Our results favor a role of NHIR in degenerating retinal neurons rather than blocking their generation as all retinal cell types were present at P6 (Fig. 5). Further experiments are required to analyze quantitatively retinal neurons differentiation in NHIR. In one third of the eyes, the peripheral retina showed advanced structural disorganization with folds and rosettes in the outer nuclear layers, which we interpreted as evidence of developmental dysplasia. In human ROP, as well as in animal models of OIR, impairment of the function of the photoreceptors and the post-receptor retina has been well-established using full-field electroretinographic and psychophysical studies [44]. Histopathologic features described in oxygen-exposed retinas include attenuation of all inner layers of the retina [45], [46], thinning and disorganized aspect of the outer plexifom layer [47], and loss of the regular stacking of the nuclei of the ONL and appearance of vacuolization [48].

Recent data suggest that inflammatory processes may participate in the pathophysiology of ROP, since inflammatory cytokines have been found to be increased in ROP eyes [49], [50]. We here show that hyperglycemia in the newborn rat led to a strong MP/MC accumulation and activation (Fig. 7). Iba1-positive cell counts showed a 80.9% increase at the peak of hyperglycemia at postnatal day 6. Furthermore, these cells also displayed an activated morphology, with shortening of their cellular processes and enlarged and round body. Finally, *Ccl2*, *Tnfα* and *Il-1β* overexpression reflects the pro-inflammatory environment induced by the hyperglycemia. In NHIR inflammation concurs with inhibited vascularization and neuronal cell loss similar to ischemic DR and ROP. If and how microglial cell activation, macrophage recruitment and proinflammatory cytokine release influence angiogenesis and neuronal apoptosis in NHIR is currently under investigation.

We believe that NHIR provides a new model to study inflammatory cascades during retinal development and will help elucidate mechanisms of inflammation-induced damage to the eyes of premature infants. Diabetic patients, and adult rodent diabetic model, also show early retinal microglial cell activation [31], [33], [34], [35] and increased concentrations of cytokines and chemokines [51], [52], [53]. NHIR might help decipher similarities and differences in the role of inflammatory cells in retinal cell loss in diabetic retinopathies and ROP.

## Conclusion

Our model reproduces aspects of ROP (inhibited angiogenesis) and common aspects of DR and ROP (neuronal cell death and inflammation). In contrast to the OIR model, which displays little inflammation, inflammation in NHIR is a prominent feature and might help decipher the inflammatory aspect of hyperglycemia-induced neuronal and vessel abnormalities in ROP. Compared to other models of rodent diabetic retinopathy, our model has the advantage of being short, having a pronounced phenotype, and not requiring insulin treatment for animal survival.
